# Safety and efficacy of amphotericin B inhalation for Candida spp. in the respiratory tract of critically ill patients

**DOI:** 10.1186/cc13549

**Published:** 2014-03-17

**Authors:** P Van der Geest, E Dieters, B Rijnders, J Groeneveld

**Affiliations:** 1 Erasmus Medical Center, Rotterdam, the Netherlands

## Introduction

Candida spp. are increasingly isolated in the critically ill, but the clinical significance hereof is hard to establish [1]. Candida spp. colonization has been suggested as a risk factor for ventilator-associated pneumonia (VAP) [2]. The efficacy and safety of inhalational amphotericin B (AB) is unknown [3]. The hypothesis was that inhalational AB deoxycholate is a safe and effective treatment for Candida spp. colonization of the respiratory tract and thereby prevents VAP and prolonged need for mechanical ventilation.

## Methods

All patients admitted to the ICU from December 2010 to 2011 with positive Candida spp. cultures of the respiratory tract and requiring mechanical ventilation >48 hours were included. AB treatment was decided by attending intensivists. The colonization index was calculated to determine the effect of AB. The clinical pulmonary infection score (CPIS) and the lung injury score (LIS) were calculated to determine pulmonary effects of AB.

## Results

**Figure 1:**
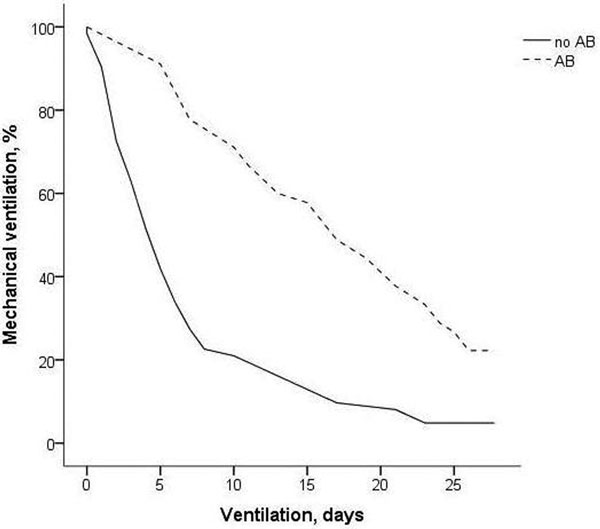
**Duration of mechanical ventilation grouped by amphotericin B**.

Fifty-five of 181 patients had been treated with AB. The AB decreased indicators of Candida spp. load but increased the duration of mechanical ventilation as compared with nontreated patients, associated with a higher CPIS and LIS, even in those with similar degree and duration of colonization and thus probably Candida spp. load at baseline (*P *< 0.001) (Figure 1). There was no difference in occurrence of VAP or mortality.

## Conclusion

AB deoxycholate treatment is effective for the treatment of Candida spp. colonization of the respiratory tract in critically ill patients. However, patients are mechanically ventilated for longer.

## References

[B1] El-Ebiary Am J Respir Crit Care Med 1997 156 583 590 10.1164/ajrccm.156.2.9612023 9279244

[B2] Azou lay E Chest 2006 129 110 117 10.1378/chest.129.1.110 16424420

[B3] Knechtel SA Expert Opin Drug Saf 2007 6 523 532 10.1517/14740338.6.5.523 17877440

